# Cost-Effectiveness of Endoscopic Stricturotomy Versus Resection Surgery for Crohn’s Disease Strictures

**DOI:** 10.3390/healthcare13151801

**Published:** 2025-07-24

**Authors:** Kate Lee Karlin, Grace Kim, Francesca Lim, Adam S. Faye, Chin Hur, Bo Shen

**Affiliations:** 1 Duke University Medical Center, 2301 Erwin Rd, Durham, NC 27710, USA; 2 Division of Digestive and Liver Diseases, New York-Presbyterian/Columbia University Irving Medical Center, 622 West 168th Street, New York, NY 10032, USA; fl2586@cumc.columbia.edu (F.L.);; 3 Division of Gastroenterology & Hepatology, New York University Langone Health, 550 First Ave, New York, NY 10016, USA

**Keywords:** Crohn’s disease, cost-effectiveness, endoscopic stricturotomy, endoscopic therapy, resection surgery

## Abstract

**Background:** Endoscopic therapies for Crohn’s disease (CD) strictures, including endoscopic balloon dilation (EBD) and endoscopic stricturotomy (ESt), are less invasive interventions compared to surgery. ESt is advantageous for strictures that are longer, more fibrotic, or adjacent to anatomic structures requiring precision, and it has shown a high rate of surgery-free survival. **Methods:** We designed a microsimulation state-transition model comparing ESt to surgical resection for CD strictures. We calculated quality-adjusted life years (QALYs) over a 10-year time horizon; secondary outcomes included costs (in 2022 USD) and incremental cost-effectiveness ratios (ICERs). We used a societal perspective to compare our strategies at a willingness-to-pay (WTP) threshold of 100,000 USD/QALY. Sensitivity analyses, both deterministic and probabilistic, were performed. **Results**: The surgery strategy cost more than 2.5 times the ESt strategy, but resulted in nine more QALYs per 100 persons. The ICER for the surgery strategy was 308,787 USD/QALY; thus, the ESt strategy was determined more cost-effective. One-way sensitivity analyses showed that quality of life after ESt as compared to that after surgery, the likelihood of repeat intervention, and surgical mortality and cost were the most influential parameters shifting cost-effectiveness. Probabilistic sensitivity analyses favored ESt in most (65.5%) iterations. **Conclusions:** Our study finds endoscopic stricturotomy to be a cost-effective strategy to manage primary or anastomotic Crohn’s disease strictures. Post-intervention quality of life and probabilities of requiring repeated interventions exert most influence on cost-effectiveness. The decision between ESt and surgery should be made considering patient and stricture characteristics, preferences, and cost-effectiveness.

## 1. Introduction

Inflammatory bowel disease (IBD) is one of the costliest gastrointestinal conditions in the United States (U.S.) [1]. A study looking at annual mean health care costs for IBD patients in the U.S. from 2007 to 2016 using administrative claims data revealed annual costs to be approximately USD 23,000, three-fold the costs for non-IBD patients [1]. For Crohn’s disease (CD), total costs in the U.S. in 2006 were estimated at between USD 10.9 and 15.5 billion [2].

Among the most common complications of CD is the development of strictures, whether primary or secondary, from post-surgical anastomosis [3,4,5,6]. Approximately 28% of patients exhibit strictures upon diagnosis, and over a third of them develop strictures within five years [7]. Strictures often are treated with surgery, which drastically increases hospitalization cost [2,8]. A retrospective cohort study found that surgical hospitalizations for CD cost approximately USD 46,354 per individual while medical hospitalizations were notably less expensive at approximately USD 20,744 [2,9].

In the last two decades, endoscopic therapies such as endoscopic stricturotomy (ESt) and endoscopic balloon dilation (EBD) have emerged as valid, minimally invasive therapeutic modalities for Crohn’s strictures [5,10]. Previously, ESt was mainly used in the treatment of upper gastrointestinal or biliary strictures [11]. Cohort studies have shown that ESt achieves comparable stricture-related surgery-free survival compared to ileocolonic resection in CD patients with primary or anastomotic strictures [12,13]. Another cohort study indicated that ESt may be more effective than EBD in treating ileocolonic primary or anastomotic strictures, with a relative decrease in the risk of perforation [5,14]. ESt offers advantages for longer, fibrotic strictures or for strictures adjacent to anatomic structures that benefit from precise incision, and it has shown 60 to 80% surgery-free survival rates at three to five years [13,14,15]. Consensus guidelines now state that ESt may be applicable to various forms of strictures, including fibrotic, anastomotic, and distal bowel strictures, particularly anorectal strictures [5,16].

Although the results of these studies have been promising, larger randomized controlled studies with longer-term follow-up are needed before stricturotomy can be recommended as one of the mainstay treatments for CD strictures [5,17,18]. Nonetheless, cost-effective analyses have the capacity to model the outcomes of intervention strategies. ESt is less costly and is associated with fewer complications than surgery, but surgery remains a primary and effective therapy for CD strictures [2,12]. Our prior cost-effectiveness model found EBD to be cost-effective over resection surgery, as surgery had an incremental cost-effectiveness ratio (ICER) of USD 113,332 per quality-adjusted life year (QALY), higher than our willingness to pay (WTP) [19]. In this study, we assessed the cost-effectiveness of ESt versus resection surgery for patients with primary or anastomotic small or large bowel strictures.

## 2. Methods

### 2.1. Base Model

The model began with a 40-year-old patient with a primary or anastomotic CD stricture, since CD patients are typically diagnosed early in life with strictures that occur around age 40 [5,12,13,14,16,20]. However, the use of biologics may alter the natural history of stricture onset moving forward [21,22,23,24]. This choice encompassed both primary and anastomotic strictures in the small or large bowel, reflecting the fact that patients with both stricture types were included in studies in the literature [12,13,14]. The patients in the cohort studies predominantly had ileal or ileocolonic strictures with stricture lengths less than 4–5 cm, making ESt feasible [12,13].

A microsimulation state-transition model was designed in TreeAge Pro Software version 2022 Williamstown, MA, USA. A microsimulation approach was used to track individuals’ unique treatment history, including procedure counts and time since last procedure [12,13]. The model incorporated two strategies, namely ESt or surgery, from which individuals would branch into different health states based on transition probabilities (Table 1) [1,2,6,7,8,9,10,11,12,13,14,15,16,17,18,19,20]. Patients accumulated cost and effectiveness values as they progressed through pathways [21,22,23,24]. At terminal nodes, except in cases of death, individuals cycled back to a starting health state for every month. All-cause mortality at each cycle was taken from life tables from the Centers for Disease Control and Prevention [25].

**Figure 1:**
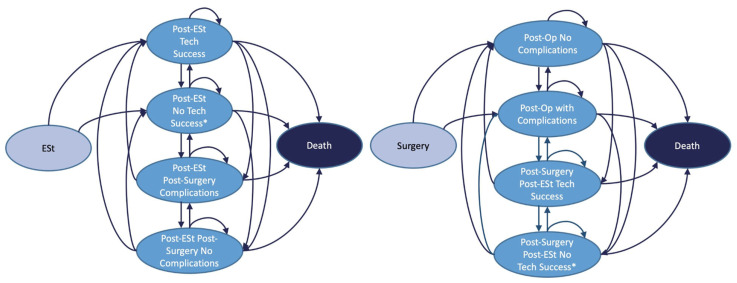
Model Schematic. * Waiting rooms were created in the model for 3- to 6-month waiting periods after an unsuccessful ESt and a subsequent ESt since, logistically, obtaining another endoscopic procedure within a month is not likely. Abbreviations: ESt (endoscopic stricturotomy), Post-Op (post-operative), Tech (technical).

A schematic of the model is provided in Figure 1. Aside from the two strategies, we did not include a null comparator, as there is no available data on the natural progression of CD strictures that could be integrated into the model. Surgical procedures included open and laparoscopic large- or small-bowel resections (CPT codes 44005–44160; 44180–44238). A societal perspective was used. Cycle length was set at one month to provide adequate but not excessive time for a given procedure and its complications. We adopted a 10-year time horizon since studies included had median follow-up periods of up to 10 years post-intervention [12,13,16]. A discount rate of 3% per year was applied to all costs and quality-adjusted life years (QALYs). A total of 100,000 patients were simulated.

### 2.2. Outcomes

The primary outcome was QALYs, rather than life-years, to take into consideration both the length of life and the quality of life. Utility values assigned to health states were drawn from the literature (Table 1) [29,30,31,32,33,34]. Effectiveness was calculated by multiplying the utility score by the time spent in each respective health state. A disutility was applied for specific events, such as surgery, in which a patient was considered to be less than their usual state of health for 4 weeks, or 8 weeks if the surgery was complicated [31,33,35].

The probabilities of undergoing ESt after ESt, ESt after surgery, surgery after ESt, and surgery after surgery were deduced by extracting data from time-to-event graphs from real-world data using Engauge Digitizer [1,2,3,12,13,14,15]. Time-to-event curves guided pathways rather than symptoms, in accordance with *Lancet* consensus guidelines [5]. We also incorporated a probability for ESt technical success. A variable was added to represent a number of endoscopic procedures that patients received before undergoing their first surgery, and that variable was varied in sensitivity analyses, in order to account for endoscopist comfort level with endoscopic therapies.

### 2.3. Costs

Costs were obtained from Medicare wherever possible and adjusted for inflation to May 2022 in U.S. dollars [26,27,36]. Productivity costs for surgery were calculated by considering the median duration of hospitalization for both uncomplicated and complicated surgeries, 9 and 16 days respectively, along with the annual mean wage data [28,37]. In the case of productivity costs for ESt, we assumed one day of lost work [28].

### 2.4. Assumptions

We assumed that all patients initiated the model with a utility value corresponding to severe CD due to their strictures. Their risk of requiring subsequent endoscopic or surgical therapy was based solely on time elapsed since the last intervention, since our model drew from best values in the literature rather than following a specific cohort. Our probabilities would represent averages from diverse cohorts. We capped the maximum number of surgeries in both strategies at 5 due to our 10-year time horizon, as it would not be likely for a patient to need more than one surgery every other year. If a patient had a technically unsuccessful ESt, we assumed that 30% of patients would try another ESt, while 70% would proceed to surgery. However, since our cycle length was one month and it is logistically not likely that a patient would be able to receive another procedure the next month, we created a 3-month waiting period for endoscopic procedures and a 6-month period for surgery, the duration of which was varied in sensitivity analyses. Since time-to-event curves in the literature often encapsulate the first few procedures, we created a factor, a decrement, to reduce probabilities of subsequent interventions.

### 2.5. Analysis

We compared the strategies using a willingness-to-pay threshold of USD 100,000 per QALY. We calculated costs, QALYs, and incremental cost-effectiveness ratios (ICERs). Sensitivity analyses evaluated uncertainty, with ranges listed in Table 1 based on confidence intervals or reported ranges in the available literature. For probabilistic sensitivity analyses (PSA), probability distributions (e.g., beta, gamma) were assigned to each parameter. The probabilistic distributions used the base case as the mean and confidence intervals or ranges from the literature to inform the standard deviation to construct a best-fitting graph of plausible values. Then, the model randomly selected values from the graphed distribution for each parameter. The PSA was run 100,000 times to assess the most preferred treatment (Table 1).

## 3. Results

### 3.1. Base Case Analysis

The surgical strategy cost USD 45,135 and the endoscopic stricturotomy strategy cost USD 16,748 (Table 2). Surgery cost USD 28,388 more than the ESt strategy but had an incremental effectiveness of 9 higher QALYs per 100 persons. The ICER of surgery was USD 308,787.03 per QALY, above the willingness to pay (WTP) threshold. The median number of ESts was 4 with an interquartile range (IQR) of 2 in the ESt strategy and 0 with IQR of 0 in the surgery strategy (Table 3). The median number of surgeries was 2 with IQR of 1 in the surgery strategy and 0 with IQR of 1 in the ESt strategy. Of individuals who initially received endoscopic stricturotomy, 32.7% underwent subsequent surgery.

Secondary endpoints assisted external validation (Table 3) [13,38]. The current literature does not provide insights into the natural progression of CD strictures with therapeutic interventions; there is also no established maximum limit for the number of ESts that patients can undergo annually [5]. The upper values in our model, 10 to 13 ESts, would be attained with a frequency of one to two ESts per year for recurring strictures [5]. Additionally, both strategies exhibited low rates of technical failure and perforation consistent with the literature.

### 3.2. Sensitivity Analyses

**Figure 2:**
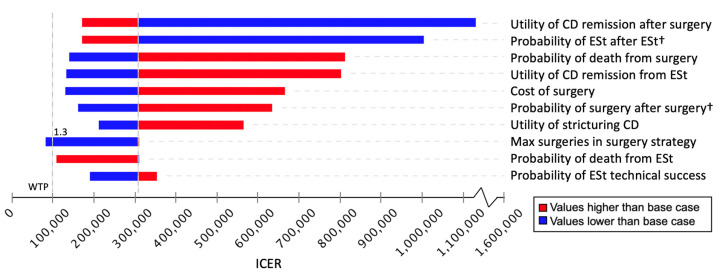
Tornado diagram showing main drivers (variables and sensitivity ranges) of the incremental cost-effectiveness ratio (ICER). † Multiplicative factor by which probability tables are multiplied. Abbreviations: CD (Crohn’s disease), ESt (endoscopic stricturotomy), ICER (incremental cost-effectiveness ratio), Max (maximum), WTP (willingness to pay).

**Figure 3:**
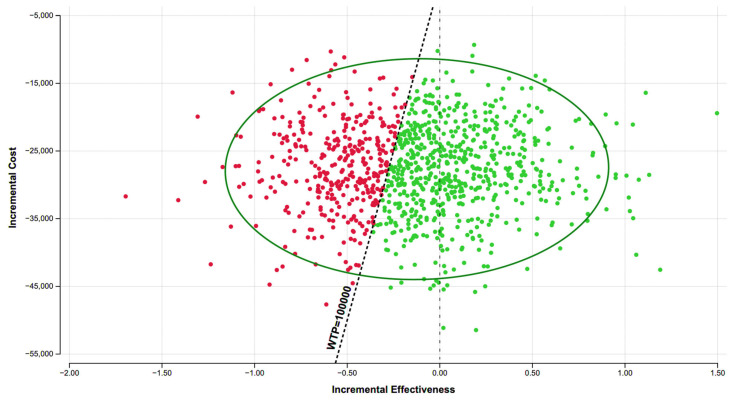
Scatterplot of probabilistic sensitivity analysis results, endoscopic stricturotomy versus surgery. Abbreviations: WTP (willingness to pay). Each dot represents one run of the probabilistic sensitivity analysis. Red dots represent instances when surgery was favored, and green dots represent instances when endoscopic stricturotomy was favored.

Sensitivity analysis showed that the most influential factors on cost and effectiveness were quality of life after intervention, probabilities of repeated interventions, and surgical mortality (Figure 2). Multivariable PSA found ESt cost-effective in 65.5% of 100,000 iterations and surgery in 34.5% of iterations when the model randomly selected a value from each parameter’s best-fitting graph of plausible values (Figure 3).

## 4. Discussion

Our study demonstrates that ESt, when feasible, is a cost-effective strategy for managing CD primary or anastomotic strictures. Although surgery accumulated greater effectiveness values, its higher cost led to an ICER of USD 308,787, higher than our willingness to pay. This adds cost-effectiveness data to support recent consensus guidelines stating that ESt may be an alternative management for certain CD strictures, especially those that are fibrotic and in close proximity to anatomic structures that require more precision, such as anorectal strictures [5,13,14,15]. Available time-to-event curves in the literature for ESt after ESt, ESt after surgery, surgery after ESt, and surgery after surgery allowed the creation of this model based on real-world probabilities of requiring subsequent intervention [12,13,14,15]. This study follows our previous cost-effectiveness model analyzing EBD versus surgical resection for primary or anastomotic CD strictures [19].

The primary outcome of quality of life showed that surgery resulted in nine more QALYs per 100 persons than ESt, supporting pre-existing knowledge that surgery is highly effective for strictures [5]. Over the 10-year horizon, however, surgery cost USD 45,135 and endoscopic stricturotomy cost USD 16,748, leading to an ICER above the WTP. It is important to note that patients who underwent ESt often had multiple ESt procedures; however, the overall cost of multiple ESts remained much lower than one surgery.

The sensitivity analysis showed that the most influential factors in altering cost-effectiveness were quality of life after intervention, the probabilities of requiring repeated ESt, and surgical mortality. This aligns with the observation that ESt often requires additional intervention. Technical success of endoscopic therapies and complication rates of both endoscopic and surgical procedures are important to consider for a given patient [11,12,13,14,16,20]. Mortality rates vary by institution, and mortality risk will differ by patient; mortality should therefore be considered when deciding the appropriate intervention for each patient [39]. The PSA results, with ESt being favored in 65.95% of iterations and surgery in the remaining 34.5%, suggest that both ESt and surgery are viable options, although ESt is more often cost-effective. ESt should therefore be considered a suitable choice for certain eligible patients in an individualized decision.

There are limitations to this study. Model-based studies inherently simplify the complexities found in real-world scenarios, where patients differ considerably with respect to their comorbidities and the characteristics of their strictures. Data was taken from cohort studies rather than randomized controlled trials, which also introduces potential biases. These cohort studies tend to be from highly specialized interventional IBD specialists, especially for ESt, which may limit broader clinical applicability; however, only highly skilled and trained IBD interventionalists would currently attempt ESt. These studies often included both primary and anastomotic strictures, making it more appropriate to model both together, although primary and anastomotic strictures can behave differently [12,13]. While we did use time-to-event data for surgery-free rates in patients treated endoscopically, we recognize that general surgical complication rates may be higher than those of patients who are eligible for endoscopic therapy, which is our base case in our model. To address this heterogeneity and uncertainty, we conducted a thorough review of published values to inform each base case input and selected a wide sensitivity range, including that of surgical complications. Since there is a lack of cost data regarding IBD surgery specifically, we used appropriate procedure codes along with Medicare and Bureau of Labor Statistics data to standardize cost inputs [19,40,41]. We did not consider other interventions for CD strictures, such as stricturoplasty or EBD, within our model, as realistically the same hypothetical patient could undergo multiple procedures during their lifetime in a microsimulation, and we wished to make purist comparisons [42]. Our results can be compared loosely to the results of our prior analysis comparing EBD and surgery [19]. Lastly, due to the lack of long-term follow-up data in the literature, we stopped our model at 10 years to limit extrapolation. Due to lack of specific utilities in the literature for the CD patient after repeated interventions, we included utilities in our sensitivity analyses as well.

**Figure 4:**
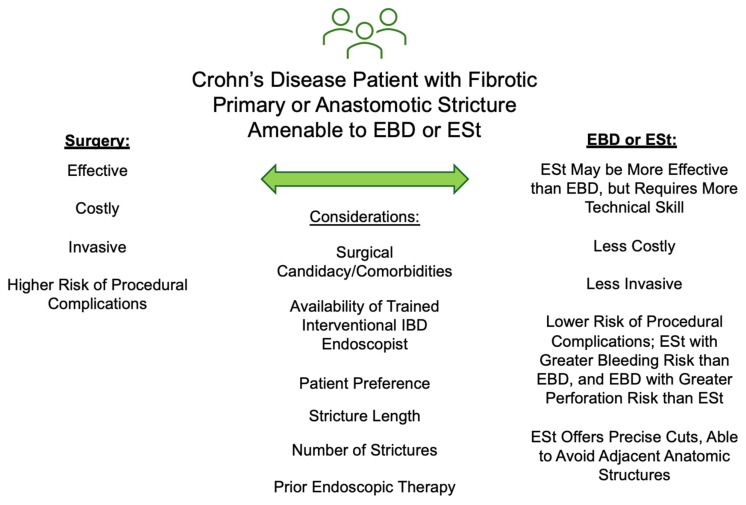
Considerations for EBD, ESt, or surgery in a CD patient. The comparisons of EBD and ESt are based on emerging data and may change with more published studies in the literature. Abbreviations: EBD (endoscopic balloon dilation), ESt (endoscopic stricturotomy), IBD (inflammatory bowel disease).

Our study has significant clinical implications. It is the first cost-effectiveness analysis investigating the use of ESt to treat primary or anastomotic strictures in CD, and it adds to practical considerations in comparing endoscopic therapy or surgery for a given CD patient (Figure 4). EST may be preferable to EBD for more fibrotic strictures, and those that abut major anatomic structures, as it allows for precise incisions [43]. The patient, stricture, and endoscopist characteristics should be considered for each patient against our model’s general base case result and PSA favoring ESt most of the time, using our deterministic analyses to shift each individual patient’s risk and benefit for surgery versus ESt. Moreover, it adds to the currently limited literature exploring surgery’s cost-effectiveness in CD.

The field of interventional IBD is advancing rapidly. There is a new study reporting the efficacy of combined surgery and intraoperative endoscopic therapy at the same time, with a one-year intervention-free rate of 82% [44]. There are additionally new studies using double balloon enteroscopy to facilitate small bowel stricturoplasties, reporting favorable efficacy [45,46]. Future cost-effectiveness analyses may incorporate multiple types of therapies and complex combinations to determine personalized CD care.

## 5. Conclusions

ESt is a cost-effective and less invasive treatment for CD primary or anastomotic strictures compared to surgery. Factors such as patient comorbidities, patient preferences, stricture characteristics, and institutional expertise in ESt should guide the decision of surgery vs. ESt for a given individual. Moving forward, longer-term and randomized controlled studies with extensive follow-up data are needed.

## Figures and Tables

**Table 1:** Input parameters.

Parameter	Base Case	Sensitivity Ranges		Distribution	References
		Low	High		
Probabilities (%)					
Technical success of endoscopic stricturotomy	0.95	0.80	1.00	Beta	[14]
Complications from endoscopic stricturotomy	0.06	0.00	0.20	Beta	[14]
Complications from endoscopic stricturotomy, perforation	0.083	0.00	1.00	Beta	[14,15]
Bleeding from endoscopic stricturotomy leading to death	0.000070	0.00	0.01	Beta	[19]
Death from endoscopic stricturotomy procedure	0.000029	0.00	0.01	Beta	[19]
Surgery after endoscopic stricturotomy with no technical success	0.70	0.00	1.00	Beta	[19]
Complications from surgery	0.21	0.088	0.43	Beta	[19]
Factor by which complications from surgery increase due to salvage surgery	1.38	1.18	1.59	Normal	[19]
Death from surgery	0.012	0.005	0.025	Beta	[19]
Death from emergency surgery	0.068	0.014	0.15	Beta	[19]
Costs ($)					
Endoscopic stricturotomy	1796.77	898.39	3593.54	Gamma	[19,26]
Surgery	23,684.73	11,842.37	47,369.46	Gamma	[19,27]
Bleeding from endoscopic stricturotomy	2338.52	1169.26	4677.04	Gamma	[19]
Productivity cost of surgery	1505.84	752.92	3011.68	Gamma	[19,28]
Productivity cost of surgical complications	1170.86	585.43	2341.72	Gamma	[19,28]
Productivity cost of endoscopic stricturotomy appointment	167.27	83.64	334.54	Gamma	[19,28]
Utilities					
Remission from endoscopic stricturotomy	0.86	0.84	0.88	Beta	[19]
Remission from surgery	0.89	0.88	0.91	Beta	[19]
Severe Crohn’s	0.62	0.50	0.73	Beta	[19]
Disutilities					
Endoscopic stricturotomy, daily x 1 day	−0.30	−0.35	−0.26	Beta	[19]
Surgery, daily, to be applied × 4 weeks	−0.22	−0.25	−0.19	Beta	[19]
Bleeding from endoscopic stricturotomy	−0.10	−0.12	−0.085	Beta	[19]
Complications from surgery	−0.24	−0.27	−0.20	Beta	[19]

**Table 2:** Base case cost-effectiveness analysis results.

	Cost ($)	Incremental Cost ($)	Effectiveness (QALY)	Incremental Effectiveness	ICER ($/QALY)
Endoscopic Stricturotomy	16,748	Reference	6.28	Reference	Reference
Resection Surgery	45,135	28,388	6.37	0.09 QALYs per person	308,787.03

Abbreviations: QALY (quality-adjusted life years), ICER (incremental cost-effectiveness ratio).

**Table 3:** Secondary endpoints.

	ESt	Surgery
Median ESts (±IQR)	4 (±2)	0 (±0)
Median surgeries (±IQR)	0 (±1)	2 (±1)
Maximum ESts (not capped)	13	10
Maximum surgeries (cap at 5)	5	5
Median ESt perforations with emergency surgery (±IQR)	0 (±0)	0 (±0)
Median failed ESts (±IQR)	0 (±0)	0 (±0)

Abbreviations: ESt (endoscopic stricturotomy), IQR (interquartile range).

## Data Availability

This study uses published data from the literature as well as public databases such as Medicare reimbursement data and U.S. Bureau of Labor Statistics, which are all available online and cited in the manuscript.

## Abbreviations

CD	Crohn’s Disease
EBD	Endoscopic Balloon Dilation
ESt	Endoscopic Stricturotomy
IBD	Inflammatory Bowel Disease
ICER	Incremental Cost-Effectiveness Ratio
PSA	Probabilistic Sensitivity Analysis
QALY	Quality-Adjusted Life Year
US	United States
WTP	Willingness To Pay
